# Characterization of VIM-1-Producing *E. coli* Isolated From a German Fattening Pig Farm by an Improved Isolation Procedure

**DOI:** 10.3389/fmicb.2019.02256

**Published:** 2019-10-01

**Authors:** A. Irrgang, B.-A. Tenhagen, N. Pauly, S. Schmoger, Annemarie Kaesbohrer, J. A. Hammerl

**Affiliations:** ^1^Unit of Epidemiology, Zoonoses and Antimicrobial Resistance, Department of Biological Safety, German Federal Institute for Risk Assessment (Bundesinstitut für Risikobewertung, BfR), Berlin, Germany; ^2^Institute for Veterinary Public Health, University of Veterinary Medicine Vienna, Vienna, Austria

**Keywords:** carbapenemase, metallo-ß-lactamase, plasmid, sequencing, chromosomally encoded, VIM-1

## Abstract

A few reports indicate that livestock might represent a new reservoir for carbapenemase-producing Enterobacteriaceae (CPE). In 2015, VIM-1-producing *Escherichia coli* were detected at slaughter in colon contents of animals from a German fattening pig farm within the national monitoring on ESBL-producing *E. coli.* In this study, pooled faces samples from pigs, as well as samples from the barn surrounding environment of this fattening farm were taken, to evaluate the dissemination of CPEs. Several modifications of the culture-dependent detection procedure were investigated for their potential to improve the sensitivity of the CPE isolation method. The current reference procedure was adapted by adding a real-time PCR pre-screening and additional enrichment steps. It was possible to isolate 32 VIM-1-producing *E. coli* from four fecal samples of three different barns using two serial enrichment steps in combination with real-time PCR and selective agar plates. By genetic typing, we confirmed the presence of two *E. coli* clonal lineages circulating on this particular farm: one was harboring the *bla*_VIM–__1_ on an IncHI2 plasmid while the second lineage carried the gene on the chromosome. Despite its different locations, the *bla*_VIM–__1_ gene was harbored on a class 1 integron in both clonal lineages. Whole-genome sequencing revealed that the VIM-1-carrying plasmids exhibited only slight variability in its compositions and sizes. We assume that the prevalence of CPEs in animal production in Germany and other European countries might be underestimated and there is a concern of further spread of VIM-1-producing bacteria in German livestock and food.

## Introduction

Carbapenems are broad-spectrum beta (ß)-lactams that are considered as “critically important antimicrobials” by the World Health Organization (WHO). They often represent the last treatment option in human medicine for infections with multidrug-resistant bacteria (WHO Advisory Group on Integrated Surveillance of Antimicrobial Resistance, 2017). These antibiotics inhibit the bacterial cell wall synthesis by interacting with the penicillin-binding protein (PBP). Carbapenems are not degraded by most ß-lactamases, including pAmpC and extended-spectrum ß-lactamases (ESBL). Acquired resistance to carbapenems can be mediated by carbapenem degrading enzymes (carbapenemases) or by changes in membrane permeability due to the loss of specific outer membrane porins (Miriagou et al., 2010). Carbapenemases confer resistance to almost all ß-lactams and typically are associated with further resistance genes for other antimicrobial classes, which strongly limit therapeutic options (Woodford et al., 2014). Since the genetic information for the synthesis of carbapenemases is mostly encoded on plasmids or transposons, these resistance genes are horizontally transmissible to other bacteria, which might further promote their spread (Patel and Bonomo, 2013). Carbapenemases have been mainly reported in Enterobacteriaceae, *Pseudomonas aeruginosa* and *Acinetobacter baumannii* (Cantón et al., 2012).

Carbapenem-resistant Enterobacteriaceae (CRE) are an emerging concern in the medical setting, especially in intensive health care units. Enterobacteriaceae include common (*Klebsiella pneumoniae, Escherichia coli, Salmonella enterica*) and rare human pathogens (i.e., *Proteus mirabilis, Raoultella planticola, Citrobacter freundii*) with increasing antibiotic resistance (Potter et al., 2016). Main risk factors for acquiring CRE among humans are intensive care therapies or hospital admission in the preceding 6 months, as well as previous travel outside the country of residence (Grundmann et al., 2017).

Carbapenems are not approved for use in veterinary medicine (OIE, 2015). However, carbapenemase-producing Enterobacteriaceae (CPE) were also reported from livestock, companion animals, wildlife, food and frequently also from rivers and sewage (Woodford et al., 2014; Köck et al., 2018). Nevertheless, carbapemases from non-human sources are up to now only sporadically isolated.

**FIGURE 1:**
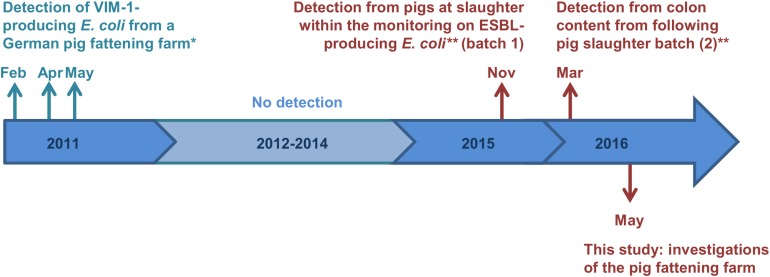
Time scale of detected VIM-1-producing *E. coli* from German pig production. Colors cyan and red indicate the two different farms, from which the samples origin. ^∗^Isolates were described previously (Falgenhauer et al., 2015; Fischer et al., 2017). ^∗∗^Study described by Irrgang et al. (2017b).

In Germany, most carbapenemases detected in human medicine are OXA-48 and VIM-1 (Robert Koch-Institut, 2018). Whereas OXA-48 and variants of it have not been detected from German livestock yet, VIM-1-producing Enterobacteriaceae (i.e., *Salmonella* Infantis and *E. coli*) were detected in chicken and pig farms in Germany occasionally since 2011 (Fischer et al., 2012, 2013). The respective *Salmonella* and *E. coli* isolates carried the *bla*_VIM–__1_ gene on IncHI2 plasmids, ranging between 300 kb and 220 kb in size, respectively (Falgenhauer et al., 2015). Due to the lack of the Tra1-transfer regions the *E. coli* plasmid was determined to be non-self-transmissible. However, apart from three deletion events, both plasmids were closely related (Borowiak et al., 2017). Almost 5 years later, VIM-1-producing *E. coli* were detected from two batches of pigs at slaughter within the German national ESBL *E. coli* monitoring (Irrgang et al., 2017b). Strikingly, isolates showed a high degree of similarity to the isolates from 2011. It is remarkable, that one of the isolates obtained from a pig at slaughter harbored the *bla*_VIM–__1_ gene and its integron within the chromosome. In Figure 1 all recovered VIM-1-producing *E. coli* isolates from Germany are summarized on a schematic time scale.

In this study, the origin of CPE found in pigs at slaughter (Irrgang et al., 2017b) was traced back to a pig fattening farm in Germany. Further investigations on the farm and the subsequent isolation of carbapenemase-producing *E. coli* from pooled fecal samples were carried out. Furthermore, the genetic relationship of the isolates in comparison to previous found VIM-1-producing *E. coli* was elucidated.

## Materials and Methods

### Sampling

The pig farm consisted of six barns for fattening pigs. Three barns were in historical buildings at the main site of the farm. The other three barns were modern identical buildings located approximately 100 m away from the old buildings. The barns were subdivided in eight to fourteen pens with thirty pigs per pen. All in all, around 2,000 animals were housed at the farm. The six barns were managed in an all in/all out system per barn. Composite fecal samples were collected from the floor at a minimum of ten different points of two opposing pens per sample resulting in 33 fecal samples. Additionally boot swab samples were taken from the alleys of each barn. Furthermore, environmental wipes were collected from trough, toys, walls, and dust.

### Isolation Procedure

All samples were tested using the method recommended by the EURL-AR for the isolation of carbapenemase-producing *E. coli* from caeca samples (Version 3; actual version can be found at: https://www.eurl-ar.eu/protocols.aspx, accessed on September 3, 2018). According to this procedure, the fecal samples were diluted 1:10 in buffered peptone water (w/v) without antimicrobial additives and pre-incubated 16–20 h at 37°C. Thereafter, this pre-enrichment culture was applied onto chromID^®^ Carba plates (Biomerieux) for selective cultivation. An aliquot of the pre-enrichment culture was stored at –80°C in glycerin.

Alternatively, a selective pre-enrichment was conducted in parallel by applying 10 g of the pooled fecal samples to 90 ml lysogeny broth (LB) supplemented with 1 mg/L cefotaxime (CTX) and plated on chromID^®^ Carba. To exclude any inhibitory effects of the chromID^®^ Carba agar plates, 100 μl of the unselective and selective pre-enrichments were additionally plated on MacConkey agar supplemented with 1 mg/L CTX.

Previous experiences had revealed that an additional selective enrichment step in LB + CTX following the unselective pre-enrichment might yield a higher recovery rate of CPEs. Thus, a loop from the glycerin stock was inoculated in LB + CTX at 37 and 44°C (Supplementary Figure S1A).

The detection of VIM-1-producing *E. coli* was supported by real-time PCR (described below), carried out from the selective enrichment broth (37°C). In case of a positive signal, individual presumptive *E. coli* colonies were picked from the corresponding MacConkey plates into LB supplemented with 0.125 mg/L meropenem for 24 h at 37°C in a 96 well microtiter plate. CPE verification was conducted by real-time PCR. *S*pecies determination of bacterial colonies was performed by MALDI-TOF MS (Biotyper, Bruker, Bremen, Germany).

### Antimicrobial Susceptibility Testing

Susceptibility to antimicrobials was determined by broth microdilution according to CLSI standard and minimal inhibitory concentration (MIC) values were interpreted according to EUCAST definitions. In case of azithromycin and temocillin tentative ECOFFS provided by EFSA were used for interpretation.

### Real-Time PCR

PCR-based detection was used to confirm the presence/absence of VIM-1 CPE in enrichment cultures. Real-time PCR was performed on a Bio-Rad CFX system. The *bla*_VIM_ primer and probe sequences were adapted from van der Zee et al. (2014) with CAL Flour^®^ Gold 540 as fluorophore and BHQ-1 as quencher (van der Zee et al., 2014). Amplification was conducted using ABsolute QPCR Mix, no ROX (Thermo Fisher Scientific) and the following conditions: initially 15 min at 95°C, 30 cycles with 15 s at 95°C and 60 s at 60°C for product amplification.

### Molecular Characterization

Pulsed-field gel electrophoresis (PFGE) was performed according to the PulseNet protocol^1^. Restriction profiles of the genomes were obtained by using *Xba*I, whereas plasmid content was analyzed using S1-nuclease. PFGE analysis and cluster analysis were conducted with BioNumerics 7.5 (Applied Maths, Sint-Martens-Latem, Belgium). To determine whether the *bla*_VIM–__1_ gene was located on plasmids, S1-PFGE gels and subsequent Southern blot hybridization following DNA–DNA hybridization were performed. For blotting, DNA from PFGE gels was transferred to a nitrocellulose membrane by vacuum blotting (1.5 h at 5 bar) with 10 × SSC. After crosslinking DNA for 2 min under UV light (312 nm), membrane was hybridized with a digoxigenin (DIG) labeled *bla*_VIM_ probe and DIG labeled Lambda DNA as positive control using DIG Easy Hyb and DIG Wash and Block Buffer Set (Roche Diagnostics, Mannheim, Germany) as recommended by the manufacturer. Transformation of *bla*_VIM–__1_-carrying plasmids was carried out by electroporation into competent *E. coli* DH10B cells (ElectroMAX^TM^ DH10B^TM^ Cells; Invitrogen^TM^, Thermo Fisher Scientific, Schwerte, Germany).

### Sequencing and Bioinformatic Analyses

Whole-genome sequencing (WGS) of selected isolates (*n* = 10) was conducted on a Miseq benchtop platform (Illumina, CA, United States) with 2 × 251 bp paired-end reads. Isolates representing different clonal lineages and/or plasmid profiles were chosen for WGS. *De novo* genome assembling was carried out using CLC Genome workbench 9.5.2 (Qiagen Bioinformatics) or PATRIC database^2^. Sequences were deposited at NCBI with the accession numbers: RYCV00000000 (R1176), RYCW00000000 (R1180), RYCX00000000 (R1182), RYCY00000000 (R1183), RYDA00000000 (R1184), RYDA 00000000 (R1191), RYDB00000000 (R1203), RYDC00000000 (R1207), RYDD00000000 (R1208), and RYDE00000000 (R1209). Information on MLST-type, plasmid content, pMLST and acquired resistance genes was obtained from sequencing data using the web-based tools provided by the Center for Genomic Epidemiology^3^. For single nucleotide polymorphism (SNP) whole-genome data were processed and interpreted using BioNumerics 7.5 (Applied Maths). Raw reads first underwent a length trimming (settings: 300 bases; minimum sequence length: 20, maximum homopolymer length: 20 bases), followed by a quality trimming (minimum quality: 5; average quality: 20). For SNP analysis SNP filters for absolute coverage, relative coverage, unreliable bases, ambiguous bases, non-informative SNPs and inter-SNP distance were applied with default parameters (Irrgang et al., 2018). Plasmid related sequences were mapped using BRIG software (Alikhan et al., 2011).

### Ethics Statement

According to the national law, permission from an ethical committee for this study is not needed. The samples were taken from the floor of the boxes. The animals of the farm were not tested directly.

## Results and Discussion

### Isolation of VIM-1-Producing *E. coli*

The pig farm was investigated in May 2016, 6 month after the first detection of a VIM-1-producing *E. coli* from the colon content of a fattening pig at slaughter (Irrgang et al., 2017b). Using the EURL-AR reference method for isolation of CPEs, no carbapenemase-producing *E. coli* were visually detected in fecal and environmental samples. However, most of the agar plates exhibited complex lawns of *Pseudomonas* bacteria that impede reliable detection of CPEs.

In contrast to the EURL-AR reference method, CPE detection using the selective enrichment with LB + CTX and subsequent plating on ChromID^®^ Carba we recovered VIM-1-producing *E. coli* isolates R1182 to R1184 from one of the samples (B6S3, sample No. 3 of barn No. 6; see Table 1). This indicated that we might have missed further positive samples due to the extensive growth of accompanying bacterial flora (i.e., *Pseudomonas*) on most of the ChromID^®^ Carba plates. Thus, a loop of the stored glycerin stocks of the unselective enrichment was transferred into LB + CTX for selective enrichment. By real-time PCR using bacteria of the selective enrichment as template *bla*_VIM_ was identified in three of the samples (B1S1, B5S5 and B6S6). In contrast to the samples B1S1, B5S5 and B6S6, the sampleB6S3 could not be detected by real-time PCR but was positive by cultivation. From the *bla*_VIM_ positive samples individual colonies were randomly taken from the corresponding MacConkey + CTX plates and sub-cultured in LB supplemented with 0.125 mg/L meropenem in 96-well microtiter plates. Bacterial growth was observed in most of the wells of samples B5S5 and B6S6. Encoded *bla*_VIM_ could be confirmed by real-time PCR.

**TABLE 1:** Summary of the VIM-1-producing *E. coli* isolates detected from fecal samples of pigs from a German fattening farm and their basic characteristics.

**Isolate**	**Barn No.**	**Sample No.**	**Beta-lactamases**	**Localization *bla*_VIM–__1_**	**Size and incompatibility group of VIM-1 plasmid**	**Resistance profile phenotype/genotype^∗^**
R1182¤	6	3	VIM-1, ACC-1, TEM-206	Plasmid	375 kb; IncHI2	ß-lactams^∗∗^, AZI, CHL, SMX, TMP/ *aadA2*, *aph(6)-Id, aph(3”)-Ib, bla*_ACC–__1_, *bla*_VIM–__1_, *bla*_TEM_, *cmlA1*, *dfrA8*, *dfrA12, mef(C)^∗∗∗^*, *mph(G), sul1*, *sul2*
R1183¤	6	3	VIM-1, ACC-1, TEM-206	Plasmid	208 kb; IncHI2	ß-lactams^∗∗^, AZI, CHL, SMX, TMP/, *aadA1, aadA2*, *aph(6)-Id*, *bla*_ACC–__1_, *bla*_VIM–__1_, *bla*_TEM_, *cmlA1^∗∗∗^, dfrA8, dfrA12, mef(C)^∗∗∗^*, *mph(G), strA, sul1, sul2*,
R1184¤	6	3	VIM-1, ACC-1, TEM-206	Plasmid	207 kb; IncHI2	ß-lactams^∗∗^, AZI, CHL, SMX, TMP/*aadA1, aadA2*, *aph(6)-Id, aph(3”)-Ib*, *bla*_ACC–__1_, *bla*_VIM–__1_, *bla*_TEM_, *cmlA1*^∗∗∗^, *dfrA8*, *dfrA12*, *mef(C)^∗∗∗^*, *mph(G)*, *sul1, sul2*,
R1185	6	6	VIM-1	Chromosomal		ß-lactams^∗∗^, SMX
R1186	6	6	VIM-1, TEM-1	Chromosomal		ß-lactams^∗∗^, CHL, SMX, TMP
R1187	6	6	VIM-1, TEM-1	Chromosomal		ß-lactams^∗∗^, CHL, SMX, TMP
R1188	6	6	VIM-1	Chromosomal		ß-lactams^∗∗^, SMX
R1189	6	6	VIM-1, TEM-1	Chromosomal		ß-lactams^∗∗^, CHL, SMX, TMP
R1190	6	6	VIM-1, TEM-1	Chromosomal		ß-lactams^∗∗^, CHL, SMX, TMP
R1191¤	6	6	VIM-1, TEM-1	Chromosomal		ß-lactams^∗∗^, CHL, SMX, TMP/*aadA1, aadA2*, *bla*_TEM–__1__*B*_, *bla*_VIM–__1_, *cmlA1^∗∗∗^, dfrA8, dfrA12, mef(C)^∗∗∗^*, *mph(G)*, *strA, sul1*^∗∗∗^, *sul2*
R1192	6	6	VIM-1, TEM-1	Chromosomal		ß-lactams^∗∗^, CHL, SMX, TMP
R1193	6	6	VIM-1, TEM-1	Chromosomal		ß-lactams^∗∗^, CHL, SMX, TMP
R1194	6	6	VIM-1	Chromosomal		ß-lactams^∗∗^, CHL, SMX, TMP
R1195	6	6	VIM-1, TEM-1	Chromosomal		ß-lactams^∗∗^, CHL, SMX, TMP
R1196	6	6	VIM-1, TEM-1	Chromosomal		ß-lactams^∗∗^, CHL, SMX, TMP
R1197	6	6	VIM-1	Chromosomal		ß-lactams^∗∗^, SMX
R1198	6	6	VIM-1, TEM-1	Chromosomal		ß-lactams^∗∗^, CHL, SMX, TMP
R1199	6	6	VIM-1, TEM-1	Chromosomal		ß-lactams^∗∗^, SMX
R1200	6	6	VIM-1, TEM-1	Chromosomal		ß-lactams^∗∗^, CHL, SMX, TMP
R1201	6	6	VIM-1	Chromosomal		ß-lactams^∗∗^, CHL, SMX, TMP
R1202	6	6	VIM-1, TEM-1	Chromosomal		ß-lactams^∗∗^, SMX
R1203¤	5	5	VIM-1, ACC-1, TEM-1	Plasmid	212 kb, IncHI2	ß-lactams^∗∗^, AZI, CHL, SMX, TMP/*aadA1, aadA2*, *aph(6)-Id bla*_ACC–__1__,_ *bla*_TEM–__1__*B*_, *bla*_VIM–__1_, *cmlA1^∗∗∗^, dfrA8, dfrA12, strA*, *mef(C)^∗∗∗^*, *mph(G)*, *strA, sul1*^∗∗∗^ *sul2*
R1204	5	5	VIM-1, ACC-1, TEM-1	Plasmid	253 kb, IncHI2	ß-lactams^∗∗^, CHL, SMX, TMP
R1205	5	5	VIM-1, ACC-1	Plasmid	208 kb, IncHI2	ß-lactams^∗∗^, SMX
R1206	5	5	VIM-1, ACC-1	Plasmid	212 kb, IncHI2	ß-lactams^∗∗^, SMX
R1207¤	5	5	VIM-1, ACC-1	Plasmid	238 kb, IncHI2	ß-lactams^∗∗^, SMX, *aadA1, aph(6)-Id, bla*_ACC–__1_, *bla*_VIM–__1_, *strA, sul1^∗∗∗^, sul2*
R1208¤	5	5	VIM-1, ACC-1, TEM-1	Plasmid	210 kb, IncHI2	ß-lactams^∗∗^, AZI, CHL, SMX, TMP/*aadA1, aadA2*, *aph(6)-Id*, *bla*_ACC–__1__,_ *bla*_TEM–__1__*B*_, *bla*_VIM–__1_, c*mlA1^∗∗∗^, dfrA12, strA, sul1^∗∗∗^*
R1209¤	1	1	VIM-1, TEM-1, TEM-206	Chromosomal		ß-lactams^∗∗^, AZI, CHL, SMX, TMP/*aadA1, aadA2*, *aph(6)-Id*, *bla*_ACC–__1__,_ *bla*_TEM–__206_, *bla*_VIM–__1_, c*mlA1^∗∗∗^, dfrA12, mef(C)^∗∗∗^*, *mph(G), strA, sul1, sul2*
R1210	1	1	VIM-1, TEM-1, TEM-206	Chromosomal		ß-lactams^∗∗^, CHL, SMX, TMP
R1211	1	1	VIM-1, TEM-1, TEM-206	Chromosomal		ß-lactams^∗∗^, CHL, SMX, TMP
R1212	1	1	VIM-1, TEM-1, TEM-206	Chromosomal		ß-lactams^∗∗^, CHL, SMX, TMP
R1213	1	1	VIM-1, TEM-1, TEM-206	Chromosomal		ß-lactams^∗∗^, CHL, SMX, TMP

*¤ Strains sequenced. ^∗^Only genes with 100% coverage and identity of 100% included; all detected genes can be found in Supplementary Table S1. ^∗∗^Resistance confirmed for the ß-lactams: ampicillin, cefepime, cefotaxime, ceftazidime, cefoxitin, imipenem; for temocillin, meropenem and ertapenem, please see the Supplementary Table S1, if the strains showed a decreased susceptibility. ^∗∗∗^Identity less than 100% (>99%), included as functionality is assumed by MIC values.*

No bacterial growth was observed in wells with colonies from the third positive sample B1S1. Here, we reverted to the glycerin stock of the unselective pre-enrichment. This stock was sub-cultivated in LB + CTX at 44°C for 24 h. This inhibited growth of *P. aeruginosa* and allowed the detection of isolates from the forth sample by subsequent plating on chromID^®^ Carba plates.

In total, 32 VIM-1-producing *E. coli* isolates from four fecal samples of three different barns (see Table 1) were recovered by the combination of the different procedures. This indicates a spread of VIM-1-producing *E. coli* within the farm but at a very low level. Boot swab samples as well as wipe samples from the environment were analyzed in a similar way to the fecal samples. For wipe samples the parallel enrichment step in LB + CTX at 37°C was dropped because the sample was not divisible. All of them were negative for CPE. There was no detection of *Salmonella* sp. from the samples, although there was a VIM-positive *Salmonella* strain isolated from a diseased pig 2 months before from the same farm (Borowiak et al., 2017).

Failure to identify positive samples using the reference method showed, that the sensitivity of this procedure is not sufficient to detect CPEs with a low prevalence in such samples. This was mostly due to the overgrowth by *Pseudomonas* sp. that are frequently intrinsic resistant to carbapenems (Livermore, 2001). In this study, a second selective enrichment step at 44°C was useful to suppress the growth of *Pseudomonas*, but Enterobacteriaceae other than *E. coli* might be also inhibited.

The current used EURL reference method is based on isolation with an appropriate selective medium. The sensitivity and specificity of commercially available media differs a lot between different studies (Aguirre-Quiñonero and Martínez-Martínez, 2017). For example sensitivity ranged from 33.3 to 100% and specificity from 54.1 to 98.9% for chromID^®^ Carba, which was used in this study. So, a combination of microbiologic and molecular methods might improve the sensitivity for the detection of CPEs.

For research purpose, analyzing the enrichment cultures by (real-time) PCR for the presence of respective genes is suitable. However, such a molecular screening of the samples is limited to the detection of some carbapenemase groups, which are depending on the established (multiplex) PCR. In our case we focus on the most prevalent carbapenemase groups (NDM, VIM, OXA-48, KPC and GES). Other or novel carbapenem genes would not be detected by routine. For monitoring programs according to the guideline 2013/652/EU it might be suitable to use two serial enrichment steps (Supplementary Figure S1B): first an unselective pre-enrichment step in buffered peptone water, using this culture for plating on selective OXA-48 media. In a second step, cultivating an inoculum of the unselective pre-enrichment culture in LB supplemented with 1 mg/L CTX at 44°C (for isolation of *E. coli*) or 37°C (for Enterobacteriaceae in general). As the suggested protocol is derived only from isolation of *bla*_VIM–__1_-producing *E. coli*, further investigations are needed, to clarify its suitability for other CPEs and other carbapenemase genes. Nevertheless, the protocol recommended by EURL-AR is used from the laboratories of the federal states at the moment, so an underestimation of the occurrence of carbapenemases in German livestock is assumed.

### Molecular Characteristics of the VIM-1-Producing *E. coli* From German Fattening Pigs

These isolates were further characterized and compared with the isolates obtained from colon samples and isolates obtained from a German pig farm in 2011 as given in Figure 1 (Fischer et al., 2017).

**FIGURE 2:**
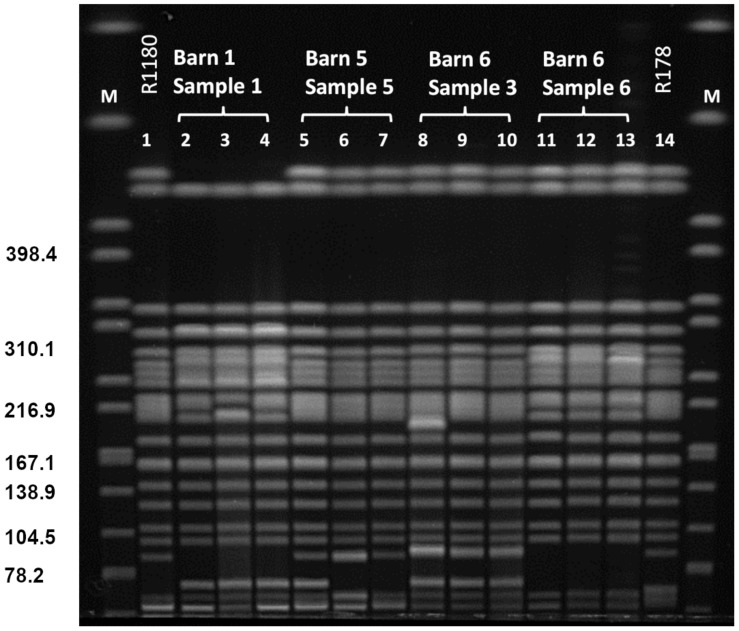
PFGE *Xba*I restriction pattern of VIM-1 producing *E. coli* from pig samples. PFGE conditions: 2.2–54.2 s; 20 h; 14°C; 6 V/cm. Lanes: 1: R1180 (Irrgang et al., 2017b); 2: R1209; 3: R1210; 4: R1211; 5: R1203; 6: R1207; 7: R1208; 8: R1182; 9: R1283; 10: R1284; 11: R1186; 12: R1191; 13: 1197; 14: R178 (Fischer et al., 2012); M: S. Braenderup H9812.

**FIGURE 3:**
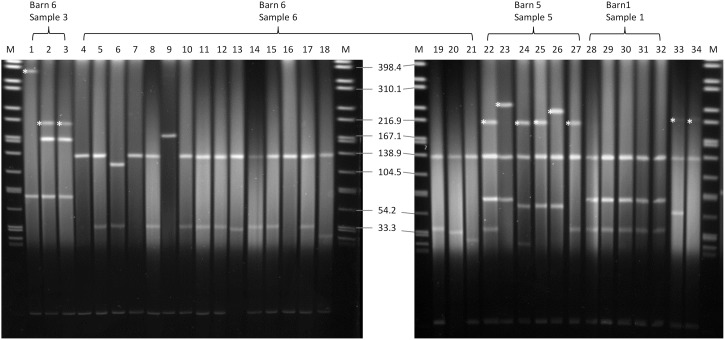
S1-nuclease PFGE. Running conditions: 1–25 s; 6 V/cm, 120°, 14°C. *bla*_VIM–1_-harboring plasmids are marked with an asterisk. 1: R1182; 2: R1183; 3: R1184; 4: R1185; 5: R1186; 6: R1187; 7: R1188; 8: R1189; 9: R1190; 10: R1191; 11: R1192; 12: R1193; 13: R1194; 14: R1195; 15: R1196; 16: R1197; 17: R1198; 18: R1199; 19: R1200; 20: R1201; 21: R1202; 22: R1203; 23: R1204; 24: R1205; 25: R1206; 26: R1207; 27: R1208; 28: R1209; 29: R1210; 30: R1211; 31: R1212; 32: R1213; 33: R178 (Fischer et al., 2012); 34: R29 (Fischer et al., 2012); M: S. Braenderup H9812.

*Xba*I-PFGE profiles of the obtained isolates showed great similarities (Figure 2). Minor differences between the isolates are based on variations in the plasmid contents (Figure 3). The *Xba*I-PFGE pattern revealed also a close phylogenetic relationship to the isolates from colon content and to the ones, which were detected in 2011 (Figure 2) (Fischer et al., 2017; Irrgang et al., 2017b). In concordance to the PFGE analyses, all isolates belonged to the multi-locus sequence type ST-88 that was also identified for the previously characterized VIM-1-producing *E. coli*. DNA–DNA hybridization of blotted S1-PFGE gels indicated that the *bla*_VIM–__1_ gene was located on plasmids representing sizes of more than 200 kb. Respective plasmids were shown in isolates from two of the samples (R1182-1184 from sample B6S3, R1203-R1208 from sample B5S5), whereas the isolates from the other two samples (B1S1, B6S6) harbored the gene on the chromosome. This indicates the spread of two very similar clonal lineages within the farm. Transformation of the *bla*_VIM–__1_ plasmid by electroporation with subsequent replicon typing confirmed that these plasmids belong to IncHI2. *bla*_VIM–__1_ carrying plasmids detected in the past from *Salmonella* and *E. coli* isolates of samples from pig production were typed equally (Fischer et al., 2013; Irrgang et al., 2017b). Interestingly, the content of plasmids among the analyzed isolates showed a high degree of variation, indicating a great plasticity in the mobilome of the VIM-1-producing *E. coli*. All isolates harbored a 135 kb IncFII/FIB plasmid (pMLST [2:A-:1]). In some of the isolates this plasmid was fused with a ∼35 kb IncX1 plasmid, which was harbored frequently. Isolate R1882 harbored a huge plasmid, containing loci of IncHI2, IncF and IncX1 plasmids, suggesting a fusion of these plasmids. It is questionable, whether plasmid fusion was original or an artifact from isolation procedure. An additional ∼70 kb plasmid could be found in some of the isolates. It is possible that due to the formation of plasmid co-integrates the properties and host range of these mobile elements might be changed (Papagiannitsis et al., 2013). The IncH12 plasmids described here are non-conjugative variants of wide-host range plasmids. Co-integration with a plasmid of incompatibility group P, L/M, N, R, W or U could make the plasmid conjugative again with an extend of the host range to a broad spectrum of bacterial species (Rozwandowicz et al., 2018). It was even shown, that co-integration sometimes only takes place for the time of transmission and plasmids separate again in the new host (Xie et al., 2018). With this strategy, localization of the *bla*_VIM–__1_ gene on a non-conjugative plasmid does not limit its spread.

Analysis of the stability of the *bla*_VIM–__1_ plasmid was carried out by subcultivation of selected strains without antibiotic pressure for at least 500 bacterial generations (>50 passages). Quantification of bacteria under selective and non-selective conditions revealed that the plasmid was highly stable in *E. coli* (data not shown). This is in concordance to the repeated isolation of the clones over a period of 6 months at one farm and also with the detection of similar *E. coli* from different farms 5 years ago (Fischer et al., 2017; Irrgang et al., 2017b). This stability concerns in regard of a further spread of VIM-1-producing *E. coli* within the German livestock.

As described before, based on the *bla*_VIM–__1_ localization two clonal lineages were detected. One clonal lineage harbored the *bla*_VIM–__1_ gene on plasmids and in the other clonal lineage it was chromosomally located. In one barn we detected both clonal lineages in different samples. Regardless its localization within the genome, the *bla*_VIM–__1_ gene was located on a class 1 integron as described (Falgenhauer et al., 2015). As the *bla*_VIM–__1_-carrying plasmids are not self-transmissible, transmission of the gene is only possible through mobilization of the integron or the plasmid. The chromosomal localization of the *bla*_VIM–__1_ gene is unique for isolates obtained from this farm. None of the VIM-1-producing Enterobacteriaceae isolated in Germany before, harbored the gene on the chromosome (Fischer et al., 2012, 2013; Roschanski et al., 2017). Integration in the chromosome provides the advantage, that the resistance determinants will be vertically transmitted stable without antimicrobial pressure whereas it remains transmissible through its integron localization. Such a transmission was shown for chromosomal located *bla*_VIM_ in *Aeromonas caviae* from Israel (Adler et al., 2014). The detection of the plasmid-mediated AmpC β-lactamase AAC-1 was a suitable marker for the localization of the VIM-1 integron as it was only present on strains harboring the *bla*_VIM–__1_ gene on a plasmid.

**FIGURE 4:**
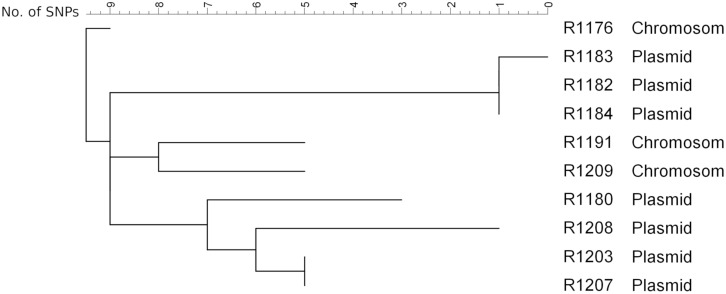
SNP analysis of the isolates obtained from the pig farm (R1182-1209) in comparison with the isolates R1176 and R1180 detected from pigs from two slaughter batches (Irrgang et al., 2017b). As reference R1184 sequenced by PacBio RS2 (to be published elsewhere) was used. Clustering was calculated using Neighbor Joining method.

To get a deeper insight into the genome and to determine the integrated sequence length, a subset of eight isolates from the farm and two isolates from slaughter samples (R1176 and R1180; Irrgang et al., 2017b) was sequenced on an Illumina Miseq. The SNP analysis of the sequence data of the isolates (mobilome excluded) confirmed the close phylogenetic relationship of the isolates (Figure 4). The isolates exhibited slight variations ranging between 0 and 17 SNPs to each other. This result suggested that all isolates originating from a common ancestor. Similar results are observed using wgMLST for phylogenetic comparison (data not shown). By SNP analysis, isolates carrying the *bla*_VIM–__1_ on the chromosome are phylogenetically slightly distinct from the ones harboring the gene on the plasmid. In addition, isolates from the same barn form separate clusters. The method was found to be robust as there were no differences observed using different reference genome for cluster analysis. It is also in concordance to cluster analysis of the *Xba*I-PFGE (Supplementary Figure S2).

**FIGURE 5:**
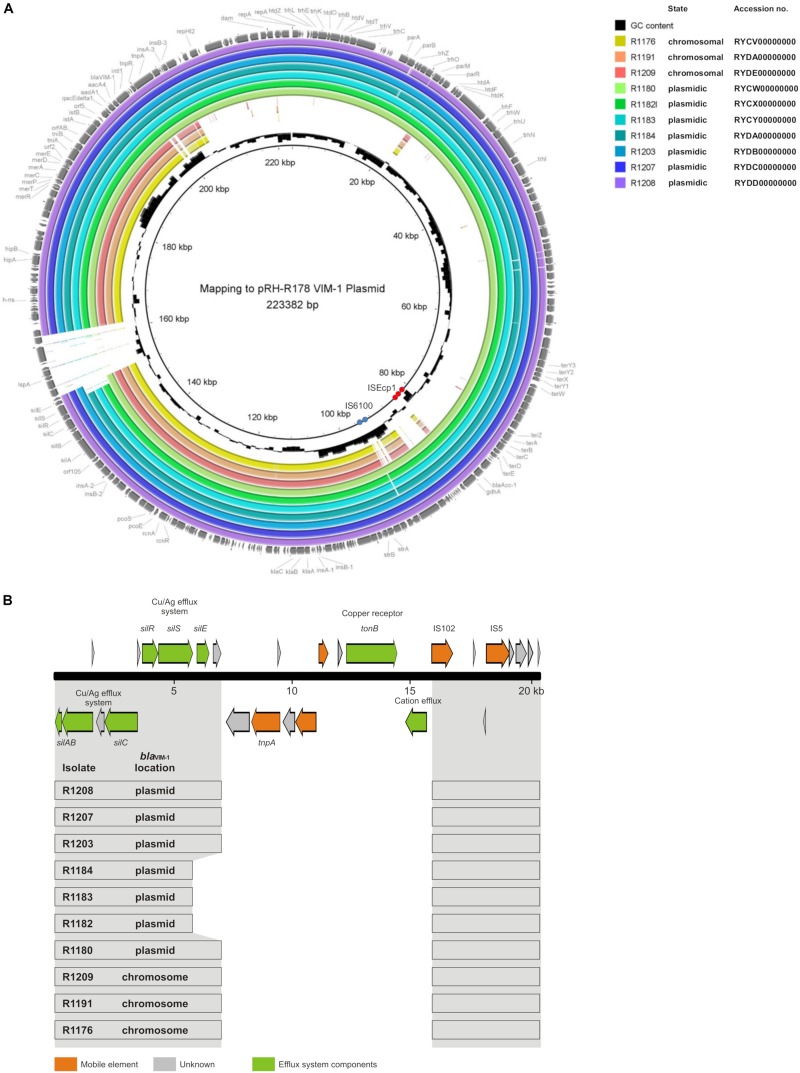
**(A)** Mapping of whole genome sequences to plasmid pRH-R178 (Acc.-No: HG530658.1) described by Falgenhauer et al. (2015). Rings 1–10 named in the legend are numbered from in to out. Rings 1–3 *bla*_VIM–1_ located on the chromosome; rings 4–10 gene located on IncHI2 plasmid. The position of repetitive sequences associated with the insertion elements IS6100 and ISEcp1 are indicated by blue and red circles, respectively. **(B)** Schematic illustration of the pRH-R178 region that is deleted in the isolates of this study. In panel **(A)**, the organization of the respective pRH-R178 region (nucleotide position 145,601–166,000) is shown. The organization of the respective pRH-R178 region (nucleotide position 145,601–166,000) is shown on the top. Genes of the specified functions are colored as designated. Below, the conserved DNA regions (gray) found in the individual isolates of this study is shown by boxes.

**FIGURE 6:**
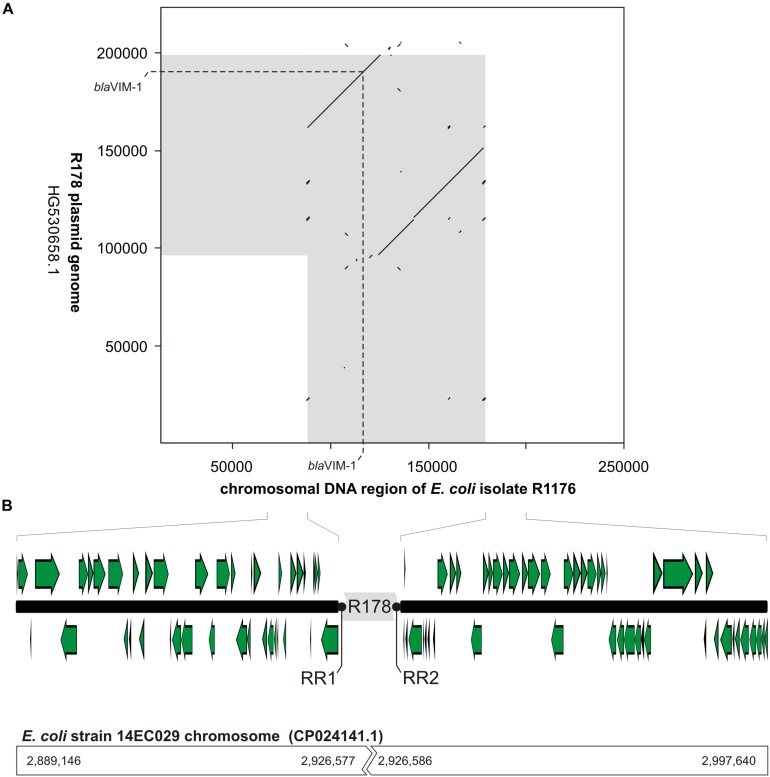
Schematic illustration of the chromosomal location of the *bla*_VIM–1_ gene in *E. coli* isolate R1176. In panel **(A)**, a dot plot alignment (minimum 85% nucleotide identity; Accelrys DS Gene, version 2.5, Accelrys Inc.) of the R178 plasmid (Accession number HG530658.1) with the respective region of the *E. coli* R1176 (RYCV00000000) chromosome is shown. DNA regions of close relationship between R178 and the R1176 chromosome are indicated in gray. Furthermore, the position of the *bla*_VIM–1_ gene is given. In panel **(B)**, the organization of the chromosomal region flanking the inserted element of R178 (gray) as well as potential target sites for the chromosomal insertion (repeat regions RR1 and RR2) are shown. In comparison to the chromosomal fragment of *E. coli* R1176 the corresponding sequence positions of the complete *E. coli* 14EC029 genome are given. While in *E. coli* R1176 approximately 100 kb of the R178 plasmid were inserted, the corresponding region of the *E. coli* isolate 14EC029 exhibited no disruption by insertion sequences.

The mapping of the sequence data to the *E. coli bla*_VIM–__1_ plasmid pRH-R178 (Acc-No.: HG530658.1) which was detected in 2011 is shown in Figure 5A. All isolates lacked a fragment of ∼10 kb length. This fragment harbored some hypothetical proteins as well as genes coding for e.g., a copper receptor, a silver-binding protein or a cation efflux pump (Figure 5B). The deleted region is flanked by a transposase upstream and might be a target of integration or excision events, which then might change the organization of this gene cluster. The rings 1–3 display sequences from the isolates harboring the *bla*_VIM–__1_ gene on the chromosome. Results indicated that a large fragment of ∼100 kb length was transferred to the chromosome. Mapping of short read WGS data to reference sequence RYCV00000000 (to be published elsewhere) of the previously characterized isolate R1176 (Irrgang et al., 2017b) indicated, that isolates detected within this study exhibited a similar chromosomal insertion of the resistance gene (Figure 6). This fragment sequence is flanked by transposases indicating that recombination events might occur frequently and location of the VIM-1 integron within the genome might change repeatedly.

### Phenotypes and Their Correlation to Genes and Plasmids

The phenotypic resistance (MIC) was determined for all isolates. Relevant phenotypes are summarized in Table 1. Further MIC values are provided in the Supplementary Material. All isolates were susceptible to tetracycline, colistin, tigecycline, (fluoro-)quinolones and gentamicin. However, resistance genes for aminoglycosides detected via WGS (*aadA*, *strA*, *strB*) indicate resistance to amikacin and/or kanamycin. In the EUVSEC layout, which is used since 2014, only gentamycin is tested. In older publications, where the former test layout was used, the correlation of the aminoglycoside resistance genes and phenotype was shown (Irrgang et al., 2017a). As seen in Supplementary Table S1 some of the strains are susceptible to meropenem and ertapenem but all strains showed MIC values above the cut-off value for imipenem. This indicates that meropenem was not the appropriate indicator antimicrobial to detect carbapenemase activity. Low MIC values for carbapenems are reported for other VIM-producing species like *A. caviae* or *K. pneumoniae* (Psichogiou et al., 2008; Adler et al., 2014). Minor variations in MIC values of cephalosporins are mostly in the range of the microdilution method. Differences in MIC could be seen for chloramphenicol, trimethoprim and azithromycin. Resistance to chloramphenicol and trimethoprim was associated with the presence of a ∼35 kb IncX1 plasmid harboring the genes *cmlA*1 (chloramphenicol resistance) and *dfrA*12/*dfrA*8 (trimethoprim resistance), respectively. The isolates R1199 and R1202 harbored a 5 kb smaller plasmid and were therefore sensitive for these two antimicrobials as well as the isolates where the plasmid was not present. In the isolates R1182, R1183, R1184, R1190 and R1204 the IncX1 plasmid seemed to be fused to the 135 kb plasmid, which is present in all other isolates, or to the *bla*_VIM–__1_-carrying plasmid. In R1207 the respective plasmid seemed to be fused to the smaller version of the IncX1 plasmid. This strain is sensitive for chloramphenicol and trimethoprim. IncX1 are narrow-host plasmids typically present in *E. coli* and *Salmonella* sp. often harboring AMR genes, especially ß-lactamases (Rozwandowicz et al., 2018). In our isolates the presence of *bla*_TEM_ genes correlates with the presence of IncX1 sequences, except for R1194 and R1201 where the plasmid was smaller indicating deletion of the gene region.

The resistance to azithromycin is related to another 70 kb plasmid, harboring *mph*(G) with 100% identity and *mef*(C) with 99.92% identity (Supplementary Table S2). This plasmid was ∼5 kb smaller in R1205, R1206 and R1207 resulting in sensitivity to azithromycin which indicated the deletion of this gene region. Based on the sequence information typing of the plasmid was not possible.

## Conclusion

In conclusion, isolation of VIM-1-producing *E. coli* from fecal samples with a low CPE load was only possible by adapting the given EURL-AR protocol with different enrichment steps in combination with a molecular screening via real-time PCR. Two closely related clonal lineages of the strain were detected, which could be pursued at the farm for at least 6 months. Persistence due to plasmid stability and vertical gene transfer of the chromosomal encoded VIM-1 carbapenemase raise the concern of further spread within the German pig production.

## Data Availability Statement

The datasets generated for this study can be found at NCBI with the accession numbers: RYCV00000000 (R1176), RYCW00000000 (R1180), RYCX00000000 (R1182), RYCY00000000 (R1183), RYDA00000000 (R1184), RYDA 00000000 (R1191), RYDB00000000 (R1203), RYDC00000000 (R1207), RYDD00000000 (R1208), and RYDE00000000 (R1209). Sequences are publically available as soon as the manuscript is published.

## Author Contributions

AI and B-AT took the samples on the farm. AI, AK, and B-AT planned and organized the study. AI, SS, and NP carried out laboratory work. AI and JH performed the bioinformatics analyses and conceptualized the study. AI, NP, and JH wrote the manuscript. All authors contributed in proofreading the manuscript.

## Conflict of Interest

The authors declare that the research was conducted in the absence of any commercial or financial relationships that could be construed as a potential conflict of interest.

## Footnotes

1 https://www.cdc.gov/pulsenet/pdf/ecoli-shigella-salmonella-pfge-protocol-508c.pdf

2 https://www.patricbrc.org/

3 http://www.genomicepidemiology.org
