# Supplementary material for: Characterization of VIM-1-Producing E. coli Isolated From a German Fattening Pig Farm by an Improved Isolation Procedure
Source: Front Microbiol. 2019 Oct 1;10:2256. doi: 10.3389/fmicb.2019.02256 (PMC6779854; doi:10.3389/fmicb.2019.02256)
Supplement: Supplementary file 3 [file Table_3.DOCX]

Supplementary Material

# Tables

# Supplement Table S1: MIC values of the isolates detected on a German fattening pig farm. Green color indicates susceptibility of the strains to the antimicrobial, red: value above the ECOFF. Data were interpreted according to EUCAST. For azithromycin and temocillin tentative ECOFFS provided by EFSA were used for interpretation.

# Supplement Table S2: Detected antimicrobial resistance genes using ResFinder 3.0.

# Figures

#
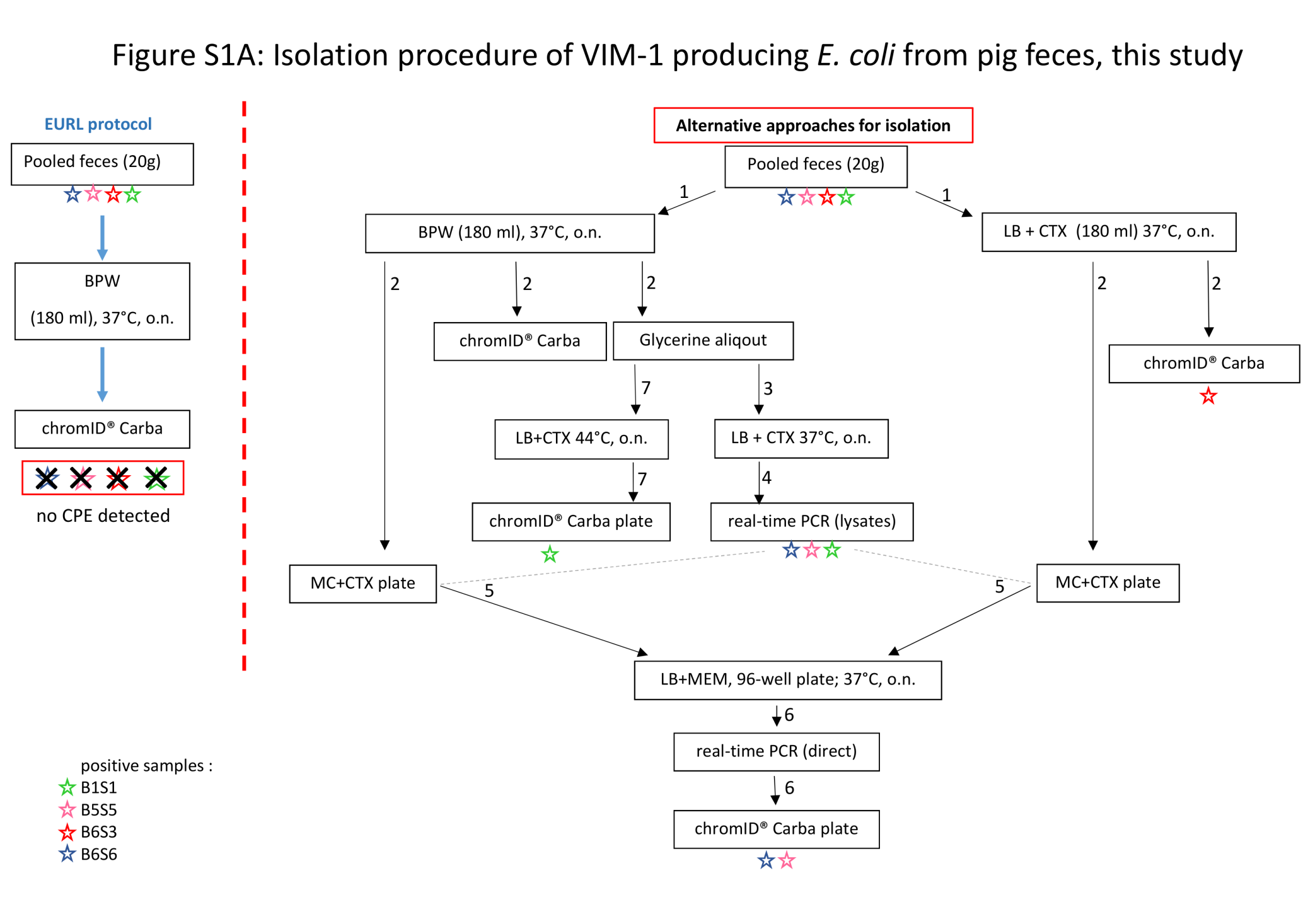


#
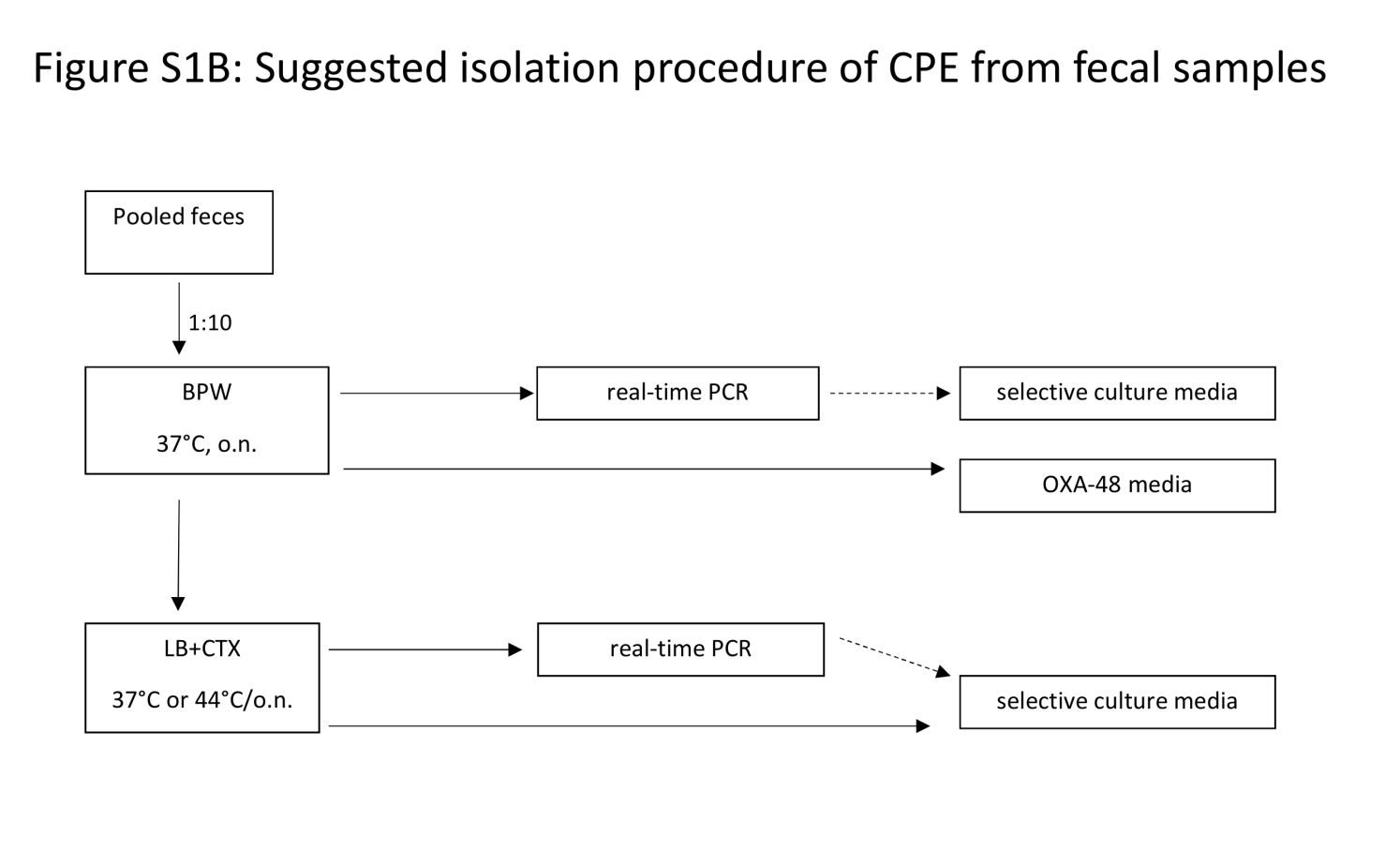


# Supplement Figure S1: A: Flow chart on the isolation procedure for detection VIM-1 producing *E. coli* from fecal samples. Numbers on the arrows indicate chronological order of isolations steps. Each color of the stars represents one of the positive samples. B: Suggested isolation procedure of CPEs or carbapenemase-producing *E. coli* from fecal samples. Abbreviations: CTX: cefotaxime, LB: lysogeny broth, MC: MacConkey agar, MEM: meropenem, BPW: buffered peptone water; o.n. overnight

#
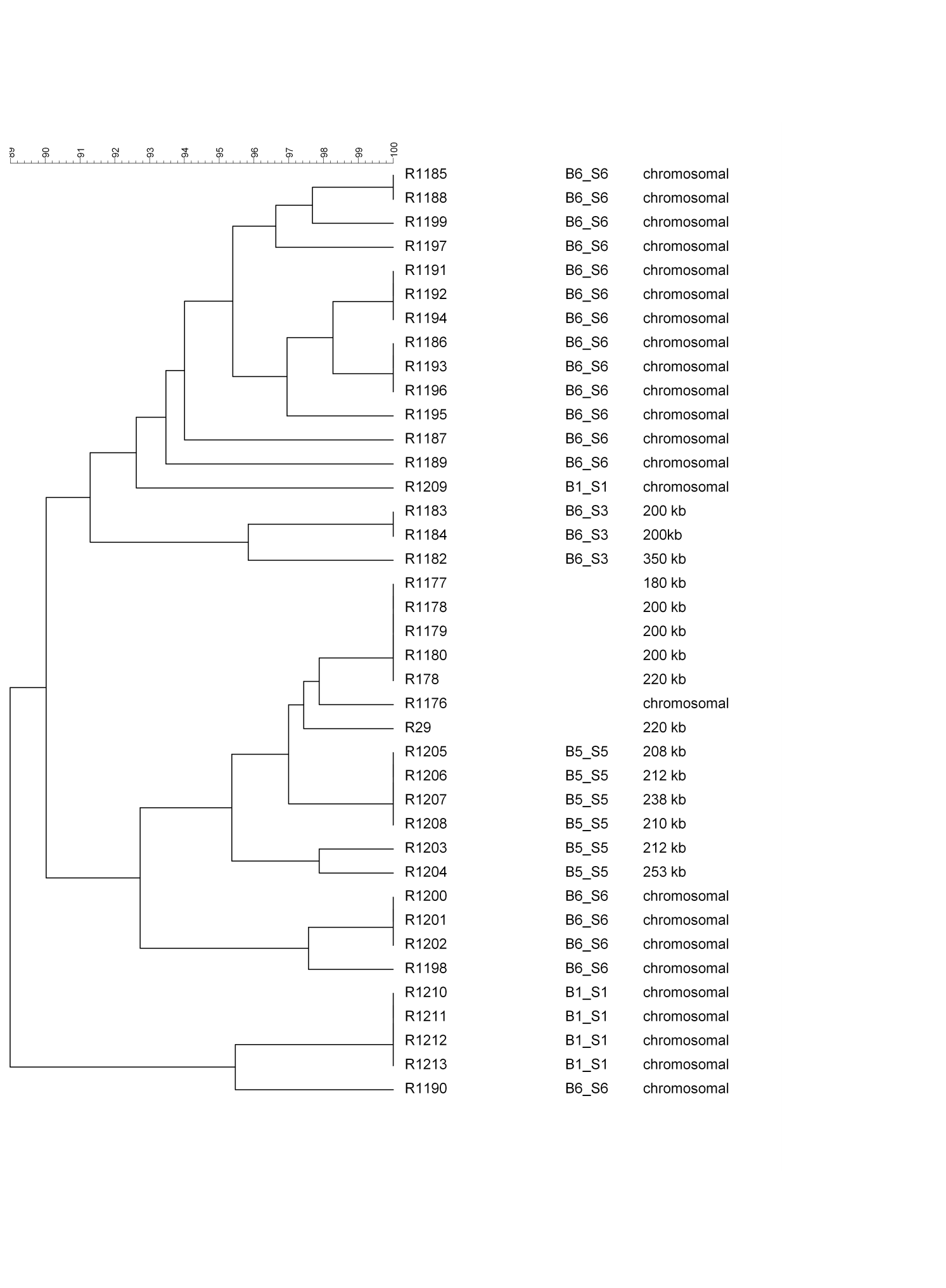


# Supplement Figure S2: Cluster analysis of XbaI PFGE from the isolates obtained by sampling of the farm compared to the isolates published before. Isolates R1176-R1180 were obtained at slaughter(Irrgang et al., 2017b); R178 and R29 were obtained from another pig farm in 2011 (Fischer et al., 2017).19 For the isolates obtained at the pig farm in this study, sample origin is provided (abbreviations B – barn no. and S – sample no.). Dendrogram was created by Bionumerics 7.6 using Dice similarity coefficient and UPGMA as algorithm for cluster calculation
